# Habitat Selection by African Buffalo (*Syncerus caffer*) in Response to Landscape-Level Fluctuations in Water Availability on Two Temporal Scales

**DOI:** 10.1371/journal.pone.0101346

**Published:** 2014-07-01

**Authors:** Emily Bennitt, Mpaphi Casper Bonyongo, Stephen Harris

**Affiliations:** 1 School of Biological Sciences, University of Bristol, Bristol, United Kingdom; 2 Okavango Research Institute, University of Botswana, Maun, Botswana; SUNY College of Environmental Science and Forestry, United States of America

## Abstract

Seasonal fluctuations in water availability cause predictable changes in the profitability of habitats in tropical ecosystems, and animals evolve adaptive behavioural and spatial responses to these fluctuations. However, stochastic changes in the distribution and abundance of surface water between years can alter resource availability at a landscape scale, causing shifts in animal behaviour. In the Okavango Delta, Botswana, a flood-pulsed ecosystem, the volume of water entering the system doubled between 2008 and 2009, creating a sudden change in the landscape. We used African buffalo (*Syncerus caffer*) to test the hypotheses that seasonal habitat selection would be related to water availability, that increased floodwater levels would decrease forage abundance and affect habitat selection, and that this would decrease buffalo resting time, reduce reproductive success and decrease body condition. Buffalo selected contrasting seasonal habitats, using habitats far from permanent water during the rainy season and seasonally-flooded habitats close to permanent water during the early and late flood seasons. The 2009 water increase reduced forage availability in seasonally-flooded habitats, removing a resource buffer used by the buffalo during the late flood season, when resources were most limited. In response, buffalo used drier habitats in 2009, although there was no significant change in the time spent moving or resting, or daily distance moved. While their reproductive success decreased in 2009, body condition increased. A protracted period of high water levels could prove detrimental to herbivores, especially to smaller-bodied species that require high quality forage. Stochastic annual fluctuations in water levels, predicted to increase as a result of anthropogenically-induced climate change, are likely to have substantial impacts on the functioning of water-driven tropical ecosystems, affecting environmental conditions within protected areas. Buffer zones around critical seasonal resources are essential to allow animals to engage in compensatory behavioural and spatial mechanisms in response to changing environmental conditions.

## Introduction

Temperate ecosystems are driven by changes in temperature, whereas tropical ones are governed by fluctuations in water availability [1]. Highly seasonal rainfall in tropical regions influences the spatial distribution of herbivores by causing temporal variation in the availability of water and the productivity of particular habitats [2]. Water availability places spatial constraints on herbivores during the dry season by forcing them to occupy habitats close to permanent water sources [3], although some species engage in long-distance central-place foraging, regularly moving between permanent water sources and foraging grounds several kilometres away [4]. During the rainy season, these spatial constraints are removed as temporary water holes are filled by rainfall, which also promotes the growth of nutrient-rich annual grasses [5].

Differences in soil type and nutrient concentration contribute to variation in the nutrient content [6] and growth rates of seasonal grasses, resulting in habitat types of disparate value [7]. Soil type influences the retention of ground water, which in turn affects habitat productivity [8]. Water availability interacts with soil type and nutrient content to affect vegetation growth, and so the distribution of particular habitat types at a landscape scale is related to their proximity to permanent water sources [9]. So seasonal changes in resource availability cause temporal changes in the profitability of a given habitat, resulting in marked seasonal patterns of habitat selection by herbivores [10]. Seasonal shifts in habitat selection often result in geographically distinct seasonal ranges, and can lead to long-distance migrations [11].

The profitability of particular habitat types can be affected by annual variation in water availability as well as seasonal cycles [12]. Such variation can introduce fluctuations on a larger temporal scale, and often cause sudden shifts in resource availability [13]. These stochastic effects can be detrimental for animals adapted to existing conditions, particularly when they are spatially-restricted by being confined to protected areas [14]. Sudden environmental changes cannot always be foreseen and, while animals are likely to engage in compensatory behaviours such as increased moving and feeding during periods of resource deficiency [15], this may not balance the effects of stochastic events [16]. Anthropogenically-induced climate change means that weather patterns are likely to become less predictable, particularly in water-governed tropical systems [1]. Annual changes in water influx into a system may interfere with seasonal cycles, affecting the behaviour of animals and potentially reducing reproductive success, and hence population health [17], [18].

The Okavango Delta in northern Botswana is a flood-pulsed ecosystem that experiences substantial seasonal and annual variations in water influx, both in terms of volume and distribution [13]. These fluctuations affect water availability, but can also alter the characteristics of habitats prone to inundation [19], and hence the spatial and temporal distribution of large herbivores. Future plans for water extraction from the Okavango River, before it enters the Delta, may compound climate-driven changes and cause sudden fluctuations in water levels on a landscape scale, with widespread implications for the ecosystem [13]. So the Okavango Delta is an ideal system to study the effects of regular and stochastic fluctuations in water availability. The regional impacts of climate change are difficult to predict, but quantifying the responses of species to existing variation will allow a greater understanding of future potential changes [1].

**Figure 1:**
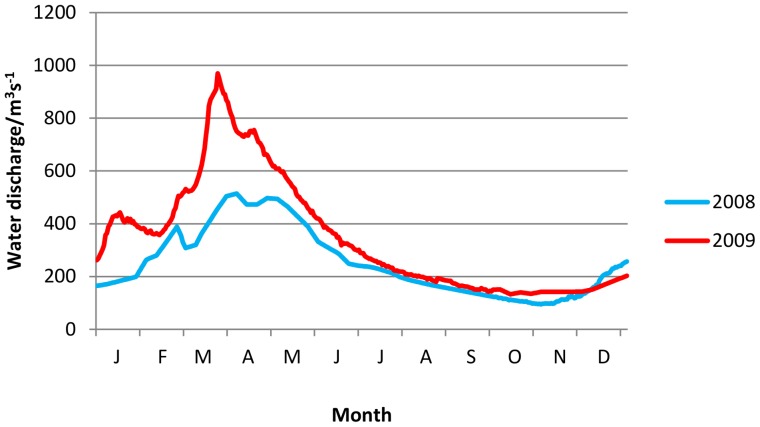
Water discharge from Okavango River between January 2008 and December 2009 at Mohembo. Redrawn with permission from data collected by the Okavango Research Institute (www.okavangodata.ub.bw).

In 2009, the volume of water entering the Okavango Delta system was almost double that in 2008 (Figure 1), thereby affecting the productivity of key foraging habitats, in particular seasonal floodplains. This could be particularly detrimental to herbivores during the late flood season, when forage in most habitat types in the Okavango Delta is at its least productive since many species rely on floodplain grasses that grow after the floodwaters recede [19]. African buffalo (*Syncerus caffer*) are among the most numerous herbivores in the Okavango Delta [20]. Being large-bodied, buffalo are capable of covering great distances in search of forage and water. While they show seasonal variations in habitat selection [21], they are water-dependent and require large quantities of forage to maintain body condition. They are therefore an ideal species to study behavioural responses to fluctuations in water levels affecting habitat productivity. We used African buffalo to test the hypotheses that (i) habitat selection varied seasonally, with habitats close to permanent water selected during the flood seasons and dry habitats further from permanent water selected during the rainy season, (ii) an increase in water levels during the late flood season affected forage availability and caused a shift in habitat selection towards permanently dry habitats, and (iii) higher water levels in 2009 reduced buffalo resting time, and caused a decrease in reproductive success and body condition during the late flood season. We use our results to examine broad issues of environmental change at the landscape level and its impact on herbivore populations in protected areas.

## Materials and Methods

### Study Area

**Figure 2:**
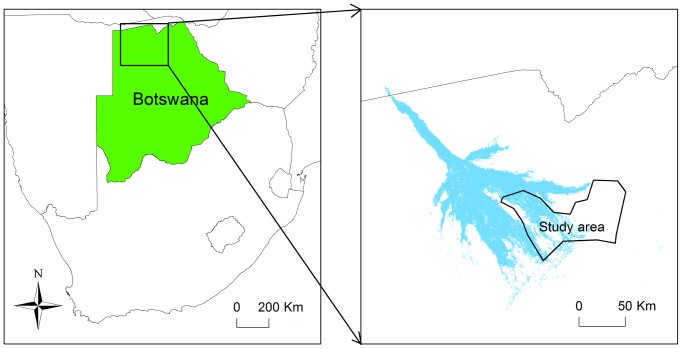
Location of study area in Botswana. The permanently flooded areas of the Okavango Delta are shown in blue in the right-hand image.

The Okavango Delta covers 15 000 km^2^ in northern Botswana, between E 22.0°–E 24.0° and S 18.5°–S 20.5° [22]. The extent of the flooded area varies seasonally, from 3 000 to 5 000 km^2^ during the driest part of the year, to 6 000 to 12 000 km^2^ during the annual flooding event, which peaks between May and August [23]. The study area was located in the south-eastern part of the Delta and included both flooded and dry regions, bounded by a veterinary fence to the south-east (Figure 2). Changing water levels were used to define three seasons: the rainy season (December to March), when most rainfall occurred; the early flood season (April to July), when flood waters were rising, and the late flood season (August to November), when flood waters were receding. Six habitat types were described, based on differences in woody and herbaceous vegetation (Table 1). Grassland occurred throughout the population range, but the other habitats were not distributed evenly across the landscape: secondary floodplain, tertiary floodplain and riparian woodland were close to permanent water channels, whereas mopane woodland and mixed acacia woodland were in areas that were dry outside the rainy season [24].

**Table 1:** Dominant species in the six habitat types found in study site.

Habitat	Dominant woody species	Dominant grass species
Secondary floodplain	None	*Panicum repens*
		*Cynodon dactylon*
Tertiary floodplain	None	*Cynodon dactylon*
		*Eragrostis rigidior*
Grassland	None	*Cynodon dactylon*
		*Dactyloctenium giganteum*
		*Eragrostis rigidior*
Riparian woodland	*Hyphaene petersiana*	*Cenchrus ciliaris*
	*Croton megalobotrys*	*Cynodon dactylon*
	*Combretum imberbe*	*Panicum maximum*
	*Lonchocarpus carpassa*	*Urochloa trichopus*
Mopane woodland	*Colophospermum mopane*	*Aristida adscencionis*
		*Urochloa trichopus*
Mixed acacia woodland	*Acacia erioloba*	*Digitaria eriantha*
	*Acacia nigrescens*	*Urochloa trichopus*
	*Terminalia sericea*	
	*Lonchocarpus nelsii*	

We produced a vector map of polygons delineating each habitat patch in the study area using geo-referenced ortho-photographs taken between 2001 and 2003 obtained from the Okavango Research Institute and manually digitised in ArcGIS 10.0 (ESRI, Redlands, CA) using a scale of 1∶10 000. Some parts of the black-and-white images had low levels of contrast, so colour images from Google Earth (Google Inc., Mountain View, CA) were used to support habitat identification. The vector map was converted to a raster map with a pixel size of 50×50 m to allow further analysis. This reduced the resolution but maintained patch distribution and accounted for errors associated with patch boundary definition. To test the accuracy of this map, we recorded 796 ground-truthing points, with a mean ± SD of 132±57 points per habitat type (range 65–224). The map represented the true habitat type 88% of the time; accuracy was lowest for grassland (79%) and highest for riparian woodland (96%).

### Capture and Collaring

Fifteen buffalo cows in different herds were fitted with Tellus Simplex 4D GPS-enabled satellite collars (Followit, Lindenberg, Sweden) programmed to record one location per hour. Cows were selected as they were more likely to retain their collars [25] and formed the core of mixed-sex breeding herds, so data from cows were representative of entire breeding herds. The collars weighed 1.8 kg, 0.4% of the weight of the smallest cow we collared (450 kg). Weight was estimated from girth measurements using a growth curve developed for buffalo in Botswana [26]. There were 24 darting operations: 15 to collar animals, two to replace malfunctioning collars and seven to remove collars. A helicopter was used for 22 darting operations; a vehicle was used twice to remove collars where the animals could be radio-tracked if visual contact was lost after darting.

Drugs used to immobilise animals were either 8 mg of A3080, reversed with Naltrexone (n = 13), or a combination of 10 mg M99, 40 mg Azaperone and 5 000 i.u. Hyalase, reversed with 42 mg M5050 (n = 11). Mean total time ± SD from darting to recovery was 15∶59±7∶28 minutes:seconds; mean time ± SD from darting to immobilisation was 4∶10±2∶24; mean time ± SD to administering the reversal agent was 10∶11±6∶17; and mean time ± SD from administering the reversal agent to being fully mobile was 1∶38±0∶55 minutes:seconds.

### Ethics Statement

One of three experienced wildlife veterinarians registered with the government of Botswana carried out each darting operation under permit from the Department of Wildlife and National Parks. All darted animals were adult females in good condition that were not obviously pregnant or with a young calf. Every effort was made to minimise the stress to darted buffalo and their herds. The helicopter remained high unless a darting sweep was being made, and we circled while waiting for the drugs to take effect so that we could maintain visual contact without disturbing the herd. The mean total time ± SD for darting sweeps was 55±14 seconds (range 31–84, n = 23). One individual had to be darted twice because she showed no effects from the drugs 20 minutes after the first dart, which had been plugged by skin as it pierced the epidermis. Although the buffalo were running during the darting sweeps, this equated to normal flight behaviour and did not cause undue distress. When a vehicle was used, the herd was followed for several hours to habituate the buffalo to our presence. They showed no signs of distress and were relaxed enough for us to approach within 60 m of the collared cow before darting. All buffalo recovered quickly from the darting operations; no ill effects were observed and they were all seen rejoining their herds.

Six collars dropped off and were recovered after the belting failed, seven animals were darted to remove collars at the end of the study, and two collars could not be recovered because they failed suddenly and ceased to emit the VHF signals by which the buffalo could be located. All capture and handling procedures were approved by the University of Bristol Ethics Committee (UB/08/034) and conformed to the American Society of Mammalogists' guidelines for the use of wild mammals in research [27]. All darting operations were carried out on government-owned protected land under control of the Department of Wildlife and National Parks, after permission had been obtained from concessionaires and all other relevant stake-holders. No protected or endangered species were involved in the research.

### Habitat Selection

Seasonal Minimum Convex Polygons (MCPs) for each individual were computed using ArcGIS 10.0 (ESRI, Redlands, CA); the first and last two weeks of GPS data from each season were omitted to ensure a clear distinction between seasons. MCPs define an animal's maximum home range size based on a polygon around its outermost known locations [28]. While MCPs can over-estimate home range size [28], they identify the area, and hence habitats, potentially available. Other methods, such as local convex hull kernel methods, are useful in identifying unused areas within a home range [29], but these may still be accessible to an animal and so should be included in calculations of habitat availability.

The adehabitatHR package [30] in R (R Development Core Team, 2008) was used to calculate the seasonal utilisation distribution (UD) for each individual via the movement-based kernel density estimation (MKDE) method [31]. This uses movement patterns derived from GPS fixes to calculate utilisation distributions, which indicate the intensity of habitat use by animals within their home ranges [32]. The minimum distance threshold (MDT), below which an animal was considered inactive, was calculated from the mean location error of each collar. Prior to deployment, each collar was hung at a height of 1 m for a minimum of 100 hours. The mean position of the fixes taken during this period was used as the reference position [33]; the distance between this and each test fix was calculated using the Point Distance tool in ArcGIS 10.0 (ESRI, Redlands, CA) and the radius of the 95% circular error probability, defined as the area containing 95% of fixes [34], was taken as the MDT. The time threshold, above which successive relocations were no longer correlated, was calculated by dividing the diameter of the MCP by ten times the median hourly distance travelled [31]. The minimum smoothing parameter was defined for each individual as the MDT plus 50 m, to account for the spread of the herd [32]. When the UDs included areas adjacent to the veterinary fence, this was identified as a fixed boundary to prevent erroneous inclusion of unavailable areas and resources [32].

Habitat selection ratios were calculated by dividing the proportion of use by the proportion of availability for each habitat [35], producing one value per season per habitat. Values were significant if their 95% confidence intervals did not include 1; those >1 indicated selection and those <1 indicated avoidance [36]. To account for the effects of scale on resource availability [37], both second and third order selection were assessed. Second order habitat selection [38] was evaluated by comparing use in the MCPs to availability in the seasonal range used by the entire population as a design III analysis [39]. Seasonal population-level MCPs were calculated from the combined relocation data from all the collared buffalo, but separate MCPs were produced for 2008 and 2009 because of the different flood levels. Third order habitat selection [38] was evaluated by comparing UD-weighted use [40] to availability in the MCPs as a design III analysis, with availability defined for each individual [39]. Using MCPs enabled us to define habitat availability at a population level by combining ranges from several individuals, as well as habitat availability in individual home ranges, allowing meaningful comparisons between the two datasets. Seasonal habitat selection ratios were subjected to Multivariate Analyses of Variance (MANOVA) to determine whether they varied significantly between seasons, and between the 2008 and 2009 late flood seasons in response to the higher water levels in 2009. Mahalanobis distances were used to check for outliers, and Pillai's trace test was used as it is robust to deviations from multivariate normality and homogeneity of variance-covariance matrices across groups [41]. Habitats where selection ratios had changed were identified using Analyses of Variance (ANOVA).

### Herbaceous Biomass

We sampled sites in each of secondary floodplain, tertiary floodplain, grassland and riparian woodland, the habitats most utilised by buffalo during the late flood season. Locations were stored on board the GPS collars and also sent via satellite to an internet server in Sweden, which emailed them to us every 10 hours. These co-ordinates were entered into a vehicle-mounted Garmin V GPS (Garmin, Schaffhausen, Switzerland) and we drove to randomly selected sites in each habitat type not rendered inaccessible by high water levels. We collected vegetation data within a 50 m radius of the co-ordinates, which allowed for the dispersion of the herd. We quantified grass biomass using a Disc Pasture Meter (DPM) [42], dropped 50 times at 1 m intervals along 5 randomly-placed 10 m transects. We avoided DPM drops on woody plants and forbs and calculated biomass as: *Y* = −1633+1791√*X*, where *X* is the mean settling height of 50 DPM drops and *Y* is the biomass in kg/ha [43]. When sites were flooded, we calculated biomass from grass cut to just below the water surface, dried in the sun and oven-dried at 60°C for 24 hours. We added the dried weights from the four quadrats and multiplied them by 10 to convert biomass from g/m^2^ to kg/ha and used a generalized linear model in R 3.0.1 to determine the effect of year on the log biomass in each of the four habitat types.

### Buffalo Movement Behaviour

We calculated the distances and turning angles between consecutive fixes taken by the GPS collars using the ‘Path, with distances and bearings’ extension (http://www.jennessent.com/downloads/Find_Path_online.pdf) in ArcView 3.2 (ESRI, Redlands, CA). Fixes ≤ MDT from the previous location were designated as resting and fixes >MDT from the previous location as active [44]. We then grouped active fixes into movement states based on their distances and turning angles using k-means cluster analysis [45]. This produced three clusters consistent with movements at different spatial scales: grazing within a patch, walking between patches, and relocating between ranges. We assigned one of these behaviours to each GPS fix, then quantified the proportion of time that buffalo allocated to each behaviour during the 2008 and 2009 late flood seasons. This compositional dataset was analysed using a multivariate analysis of variance (MANOVA) after conversion into an ‘acomp’ format using the ‘compositions’ package in R 3.0.1 [46]. The movement data from the GPS collars were used to calculate the total distance covered each day by the collared buffalo during the 2008 and 2009 late flood seasons. We ran a linear mixed model to determine the effect of year on the log of the daily distance travelled, with individual buffalo included as a random effect, using the ‘nlme’ package in R 3.0.1.

### Demographic Composition and Body Condition

We recorded the demographic composition of all buffalo herds encountered during field work, whether or not they contained collared animals. To ensure that each buffalo was only assessed once, demographic categories were recorded for a minimum of 50% of the herd as they walked past a fixed point. The horns, genitals and body size were used to classify buffalo as adult, sub-adult, juvenile and calf, with adults and sub-adults also classified as males or females [47]. Body condition of each animal was scored using a system adapted from [47] based on the visibility of the ribs and pelvis, and the presence of fat deposits on the neck and tail base (Table 2). Although subjective, such visual assessments reflect bone marrow fat content [48] and are widely used in ungulates [49]. While body condition may not be representative of an animal's health, for example if it is an asymptomatic disease carrier, changes in general body condition reflect variations in forage intake. There was no significant difference between the body condition scores (BCS) of juveniles and calves [50], so these were grouped as ‘young’, and gender only had an effect on BCS of adults, so four categories, adult male, adult female, sub-adult and young, were used for the analyses.

**Table 2:** Criteria used for determining body condition scores.

Body condition score	Description	Ribs and pelvis	Tail base	Other
1	Very poor	Prominent	Concave	Muscle wastage
2	Poor	Prominent	Concave	
3	Fair	Clearly visible	Slightly concave	
4	Good	Barely visible	Convex	
5	Excellent	Not visible	Convex	Fat rolls on neck

To assess reproductive success, generalized linear models with binomial distributions were used in R 3.0.1 to compare young:adult female and calf:adult female ratios in the two years. Adult male buffalo leave breeding herds when their body condition falls [47], so the ratios of adult males:adult females in the two years were also compared.

The counts of individual buffalo in each BCS category were analysed using a cumulative link mixed model with individual herd included as a random effect [51] to determine whether the different water levels in the 2008 and 2009 late flood seasons had an effect on buffalo body condition. This is a form of ordinal logistic regression that treats the rank order of BCS categories as a linked set of binary response variables. To compare BCS in 2008 and 2009, the model calculated the difference in the likelihood that a buffalo had a BCS of 2 rather than 1, 3 rather than 2, 4 rather than 3 and 5 rather than 4.

## Results

### Seasonal Habitat Selection

**Figure 3:**
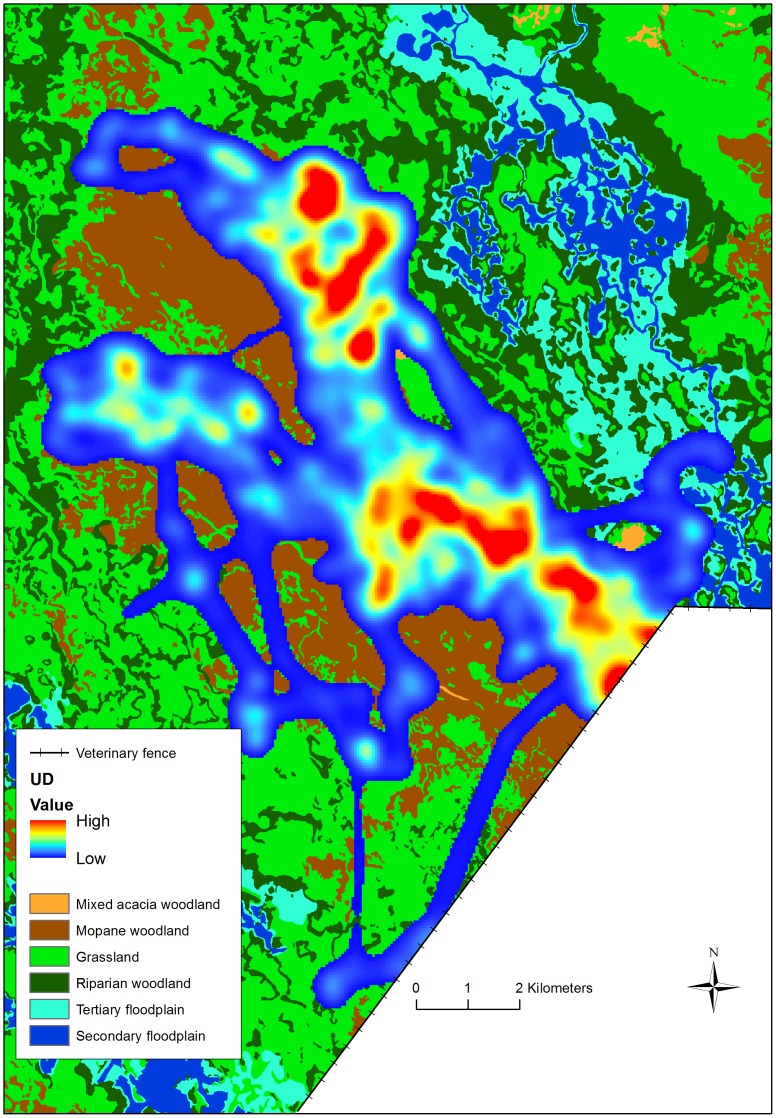
Example of a utilisation distribution produced using the Movement Density Kernel Estimation method. The figure is based on 1754 GPS fixes from one collared buffalo, B5, during the rainy season of 2009.

Seasonal UDs were produced for each collared buffalo, giving 11, 13 and 14 UDs, based on a mean ± SD of 1378±635, 1476±627 and 1476±406 GPS fixes for the early flood, late flood and rainy seasons respectively (Figure 3). Variations in collar efficiency and darting date resulted in unequal numbers of GPS fixes from each buffalo (Table 3), but using UDs meant that GPS locations were converted into intensity of habitat use, removing any potential bias associated with differential sample sizes. The MKDE method allowed use to be calculated from the GPS fixes, but also enabled the estimation of movement paths between them, and therefore provided a probabilistic measure of habitat use when fixes were not acquired, as long as the period between consecutive fixes was below the time threshold [31]. The mean ± SD values for the MDT and the time threshold were 66.1±20.3 m and 8.3±2.4 hours, respectively.

**Table 3:** Number of GPS fixes and the parameters used to calculate the utilisation distributions each season for the 15 buffalo cows.

Buffalo ID	Season	Year	MDT/m	Time/hours	Number of GPS fixes
B1	Rainy	2008	60	8	1845
B2	Rainy	2008	80	10	1028
B3	Early flood	2008	50	10	1803
	Late flood	2008	50	10	1995
	Rainy	2009	50	8	1230
B4	Early flood	2008	70	10	1804
	Late flood	2008	70	10	1774
	Rainy	2008	70	10	1282
B5	Early flood	2008	90	10	1921
	Late flood	2008	90	10	1967
	Rainy	2009	90	5	1754
B6	Early flood	2008	60	10	1751
	Late flood	2008	60	10	1793
	Rainy	2008	60	10	1400
B7	Early flood	2009	50	10	1913
	Late flood	2008	50	10	701
	Rainy	2009	50	9	1774
B8	Early flood	2009	100	10	1884
	Late flood	2008	100	6	653
	Rainy	2009	100	5	1718
B9	Early flood	2009	70	5	410
	Late flood	2009	70	10	1917
	Rainy	2010	70	5	1858
B10	Early flood	2009	50	10	430
	Late flood	2009	50	9	1174
B11	Late flood	2009	60	10	2147
	Rainy	2010	60	7	1922
B12	Early flood	2009	40	10	462
	Late flood	2009	40	10	2108
	Rainy	2010	40	4	719
B13	Early flood	2010	40	10	1285
	Late flood	2009	40	10	1756
	Rainy	2010	40	4	1827
B14	Late flood	2009	70	4	640
	Rainy	2010	70	6	819
B15	Early flood	2010	100	10	1500
	Late flood	2009	100	5	563
	Rainy	2010	100	9	1481

MDT is the minimum distance threshold below which an animal was considered inactive, and was calculated from the mean location error of each collar. The time threshold, above which successive relocations were no longer correlated, was calculated by dividing the diameter of the MCP by ten times the median hourly distance travelled.

Overall tests of second order habitat selection showed that habitat use was disproportionate to availability during the early flood (X^2^_49_ = 239.18, p<0.001), late flood (X^2^_63_ = 184.84, p<0.001) and rainy (X^2^_59_ = 296.54, p<0.001) seasons. Third order habitat selection was significant during the early (X^2^_48_ = 75.14, p = 0.007) and late flood (X^2^_59_ = 173.80, p<0.001) seasons, but not during the rainy season (X^2^_59_ = 38.74, p = 0.98). Differences in degrees of freedom were caused by the absence of some habitat types in individual MCPs. Habitat selection varied seasonally: buffalo selected mopane woodland and mixed acacia woodland during the rainy season, and avoided secondary floodplain. They selected tertiary floodplain during the late flood season, and avoided mopane woodland and mixed acacia woodland; mixed acacia woodland was also avoided during the early flood season (Table 4).

**Table 4:** Seasonal second and third order habitat selection ratios and confidence intervals.

Habitat type	Order	Habitat selection ratios (95% confidence intervals)		
		Early flood	Late flood	Rainy
		(n = 11)	(n = 13)	(n = 14)
Secondary floodplain	Second	1.29 (0.64–1.93)	1.04 (0.53–1.55)	**0.34 (0.06–0.61)**
	Third	0.97 (0.72–1.22)	1.33 (0.98–1.67)	**0.61 (0.33–0.88)**
Tertiary floodplain	Second	1.51 (0.97–2.05)	1.15 (0.71–1.59)	0.87 (0.31–1.43)
	Third	1.23 (0.97–1.49)	**1.60 (1.27–1.92)**	0.85 (0.67–1.03)
Grassland	Second	1.04 (0.81–1.27)	1.01 (0.80–1.23)	0.97 (0.72–1.22)
	Third	0.92 (0.71–1.14)	0.86 (0.63–1.08)	0.99 (0.84–1.14)
Riparian woodland	Second	1.15 (0.88–1.42)	1.10 (0.94–1.26)	0.84 (0.53–1.15)
	Third	1.08 (0.78–1.38)	1.18 (0.94–1.43)	1.00 (0.84–1.16)
Mopane woodland	Second	0.62 (0.21–1.04)	0.78 (0.43–1.13)	**1.55 (1.16**–**1.95)**
	Third	0.81 (0.40–1.22)	**0.54 (0.28–0.79)**	1.03 (0.90–1.15)
Mixed acacia woodland	Second	**0.41 (−0.04–0.86)**	1.00 (0.37–1.62)	0.99 (0.50–1.47)
	Third	1.10 (0.09–2.11)	**0.44 (0.17–0.72)**	**1.12 (1.02–1.22)**

Ratios with 95% confidence intervals that did not include 1 indicated selection (>1) or avoidance (<1) of particular habitat types. Significant results are shown in bold. Second order selection compared habitat use in individual MCP ranges to availability in the population range; third order selection compared habitat use in the utilisation distributions to availability in the individual MCPs.

MANOVAs showed significant differences between second order selection in all seasons, caused by greater selection for secondary and tertiary floodplains and riparian woodland, together with greater avoidance of mopane woodland, during the early and late flood seasons than during the rainy season (Table 5). The only significant difference in third order selection ratios was between the late flood and rainy seasons. Third order selection for secondary and tertiary floodplains was significantly greater during the late flood season, when mopane woodland and mixed acacia woodland, which were further from permanent water, were avoided.

**Table 5:** Results from MANOVAs comparing second and third order habitat selection ratios in different seasons.

Seasons	Habitat	Second order	Third order
Early flood vs. late flood	Overall	**Pillai_1,17_ = 0.552,** **p = 0.020**	Pillai_1,17_ = 0.312, p = 0.317
	Secondary floodplain	F_1,22_ = 1.008, p = 0.326	F_1,22_ = 3.478, p = 0.076
	Tertiary floodplain	F_1,22_ = 2.413, p = 0.135	**F_1,22_ = 5.200, p = 0.033**
	Grassland	F_1,22_ = 0.064, p = 0.803	F_1,22_ = 0.763, p = 0.392
	Riparian woodland	F_1,22_ = 0.616, p = 0.441	F_1,22_ = 1.078, p = 0.310
	Mopane woodland	F_1,22_ = 0.452, p = 0.509	F_1,22_ = 3.252, p = 0.085
	Mixed acacia woodland	F_1,22_ = 0.790, p = 0.384	**F_1,22_ = 8.005,** **p = 0.010**
Late flood vs. rainy	Overall	**Pillai_1,20_ = 0.467,** **p = 0.033**	**Pillai_1,20_ = 0.742,** **p<0.001**
	Secondary floodplain	**F_1,25_ = 8.922,** **p = 0.006**	**F_1,25_ = 9.572, p = 0.005**
	Tertiary floodplain	F_1,25_ = 2.911, p = 0.100	**F_1,25_ = 21.236,** **p<0.001**
	Grassland	F_1,25_ = 0.248, p = 0.623	F_1,25_ = 2.124, p = 0.158
	Riparian woodland	**F_1,25_ = 5.397, p = 0.029**	F_1,25_ = 3.824, p = 0.062
	Mopane woodland	**F_1,25_ = 17.547,** **p<0.001**	**F_1,25_ = 20.426,** **p<0.001**
	Mixed acacia woodland	F_1,25_ = 0.234, p = 0.633	**F_1,25_ = 36.589,** **p<0.001**
Rainy vs. early flood	Overall	**Pillai_1,18_ = 0.513,** **p = 0.027**	Pillai_1,18_ = 0.338, p = 0.224
	Secondary floodplain	**F_1,23_ = 15.755,** **p<0.001**	**F_1,23_ = 4.900, p = 0.037**
	Tertiary floodplain	**F_1,23_ = 9.098,** **p = 0.006**	**F_1,23_ = 9.833, p = 0.005**
	Grassland	F_1,23_ = 0.502, p = 0.486	F_1,23_ = 0.128, p = 0.724
	Riparian woodland	F_1,23_ = 6.798, p = 0.158	F_1,23_ = 0.823, p = 0.374
	Mopane woodland	**F_1,23_ = 19.025,** **p<0.001**	F_1,23_ = 1.196, p = 0.285
	Mixed acacia woodland	F_1,23_ = 1.024, p = 0.322	F_1,23_ = 0.627, p = 0.436

Significant results are shown in bold. Second order selection compared habitat use in individual MCP ranges to availability in the population range; third order selection compared habitat use in the utilisation distributions to availability in the individual MCPs.

### Annual Habitat Selection and Biomass

Annual changes in habitat selection were assessed using data from 13 buffalo, 6 collared in 2008 and 7 in 2009; two buffalo were not collared during the late flood seasons. Overall tests of second order habitat selection showed that habitat use was disproportionate to availability during the late flood season in 2008 (X^2^_29_ = 117.31, p<0.001) and 2009 (X^2^_34_ = 67.53, p<0.001). Overall third order habitat selection was also significant during the late flood season in 2008 (X^2^_25_ = 93.73, p<0.001) and 2009 (X^2^_34_ = 80.06, p<0.001). Differences in degrees of freedom were caused by the absence of some habitat types in individual MCPs. Habitat selection ratios were calculated for the late flood season in 2008 and 2009 separately (Table 6). MANOVAs showed that in 2009 there was no significant change in second order selection (Pillai_1,6_ = 0.543, p = 0.420) but there was a significant change in third order selection (Pillai_1,6_ = 6.719, p = 0.018). ANOVAs showed that there was a significant increase in the selection of grassland (F_1,11_ = 16.202, p = 0.002) and riparian woodland (F_1,11_ = 7.117, p = 0.022), although there were no differences in the selection of secondary floodplain (F_1,11_ = 1.480, p = 0.249), tertiary floodplain (F_1,11_<0.001, p = 0.983), mopane woodland (F_1,11_ = 3.106, p = 0.106) or mixed acacia woodland (F_1,11_ = 1.170, p = 0.303).

**Table 6:** Second and third order habitat selection ratios and confidence intervals during the late flood season in 2008 and 2009.

Habitat	Order	Habitat selection ratios (95% confidence intervals)	
		2008	2009
Secondary floodplain	Second	1.46 (0.55–2.36)	0.69 (0.31–1.08)
	Third	1.47 (0.90–2.04)	1.07 (0.91–1.24)
Tertiary floodplain	Second	1.50 (0.95–2.05)	0.86 (0.31–1.40)
	Third	**1.63 (1.16–2.09)**	**1.56 (1.08–2.03)**
Grassland	Second	0.95 (0.70–1.20)	1.07 (0.72–1.42)
	Third	**0.61 (0.48–0.73)**	1.05 (0.81–1.29)
Riparian woodland	Second	1.14 (0.95–1.34)	1.07 (0.81–1.33)
	Third	0.98 (0.81–1.15)	**1.37 (1.06**–**1.67)**
Mopane woodland	Second	**0.51 (0.02–0.99)**	1.01 (0.65–1.38)
	Third	**0.46 (0.22–0.71)**	**0.57 (0.20–0.93)**
Mixed acacia woodland	Second	0.56 (−0.24–1.37)	1.42 (0.67–2.16)
	Third	**0.25 (−0.10–0.60)**	**0.52 (0.19–0.85)**

Ratios with 95% confidence intervals that did not include 1 indicated selection (>1) or avoidance (<1) of particular habitat types. Significant results are shown in bold. Second order selection compared habitat use in individual MCP ranges to availability in the population range; third order selection compared habitat use in the utilisation distributions to availability in the individual MCPs.

**Figure 4:**
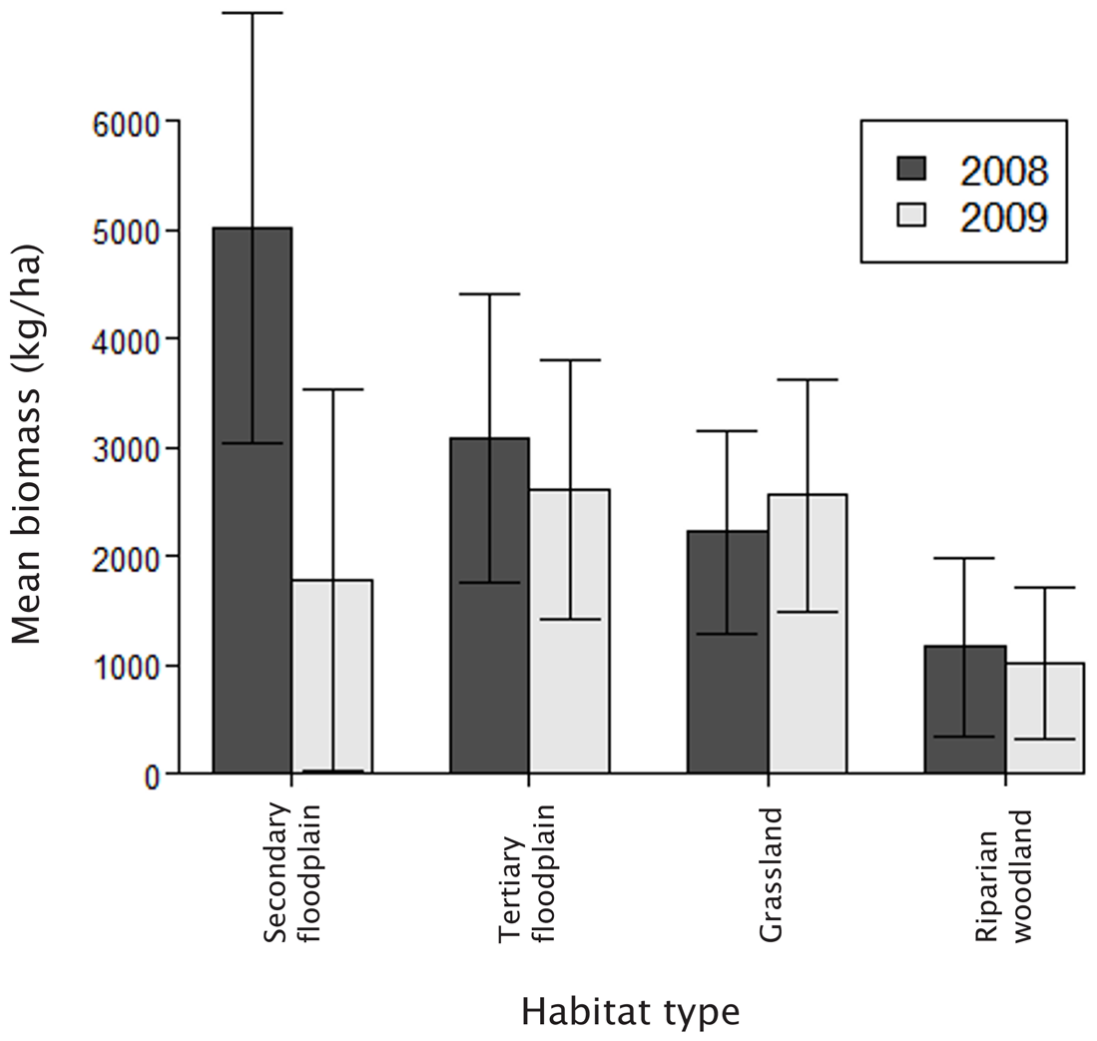
Mean herbaceous biomass in four habitats during the 2008 and 2009 late flood seasons. Error bars represent one standard deviation.

We estimated biomass at 157 and 101 sites in 2008 and 2009 respectively (Table 7). There was a significant interaction between year and habitat type (Δdeviance_3_ = 5.53, p<0.001), which was probably caused by higher biomass in secondary floodplain during the 2008 late flood season (Figure 4). Biomass was lower in both seasonally-flooded habitat types during the 2009 late flood season.

**Table 7:** Number of sites sampled for biomass in the four habitats most utilised by buffalo during the 2008 and 2009 late flood seasons.

Year	Secondary floodplain	Tertiary floodplain	Grassland	Riparian woodland
2008	36	50	37	34
2009	10	30	30	31

### Annual Changes in Behaviour, Reproductive Success and Body Condition

The clustering technique consistently identified similar distances and turning angles for each behaviour category (Table 8). Within categories, turning angles showed greater variation than distances moved, but mean turning angle reduced progressively from resting to relocating, confirming that movements over long distances were less tortuous than those over short distances. Year had no significant effect on the proportion of time spent in each behaviour (Pillai_1, 11_ = 0.548, p = 0.133) (Table 9). Buffalo travelled a mean ± SD of 7250±3551 m per day in 2008 (n = 6 buffalo, 392 days) and 7763±3821 m in 2009 (n = 7 buffalo, 483 days); the difference was not significant (LR_3_ = 1.17, p = 0.279).

**Table 8:** Distances and turning angles for the different behaviour categories for buffalo collared during the 2008 and 2009 late flood seasons.

ID	Mean distance (m) ± SD				Mean turning angle (°) ± SD			
	Rest	Graze	Walk	Relocate	Rest	Graze	Walk	Relocate
B3	22±14	190±93	559±141	1266±365	96±53	61±48	53±45	43±42
B4	22±14	200±101	629±177	1709±540	97±53	67±50	54±46	46±48
B5	31±22	258±109	689±177	1700±576	96±53	67±50	54±45	42±41
B6	25±18	233±113	709±206	1966±528	95±55	61±48	50±44	40±32
B7	20±13	208±106	636±169	1560±646	99±54	71±51	57±45	43±38
B8	31±25	272±108	679±155	1453±443	94±55	58±46	51±42	47±43
B9	22±16	247±125	760±203	1933±644	97±53	65±50	53±45	42±41
B10	19±13	194±107	666±216	2263±588	104±53	76±53	59±49	21±28
B11	16±9	204±122	692±196	1702±516	99±53	63±50	48±42	35±37
B12	18±13	210±98	579±134	1215±396	95±54	59±47	50±42	42±39
B13	14±8	173±108	623±172	1535±490	99±53	68±51	51±43	48±42
B14	24±17	256±123	725±188	1761±566	94±53	58±47	47±41	43±39
B15	33±26	269±114	741±194	1912±660	97±54	63±48	57±48	41±43

**Table 9:** Mean percentage of time spent engaging in four behaviours by buffalo during the 2008 and 2009 late flood seasons.

Year	Rest	Graze	Walk	Relocate
2008	34.7	42.3	20.2	2.8
2009	30.8	43.0	22.6	3.6

Mean ± SD young:adult female ratios in 2008 (n = 18 herds) and 2009 (n = 15 herds) were 0.478±0.183 and 0.535±0.154, respectively; a quasibinomial distribution was used to account for overdispersion but there was no significant difference between years (t_31_ = 0.983, p = 0.333). Mean ± SD calf:adult female ratios in 2008 and 2009 were 0.197±0.130 and 0.156±0.107, respectively; there was a significant difference between years (z_31_ = 3.027, p = 0.002). Mean ± SD adult male:adult female ratios in 2008 and 2009 were 0.434±0.298 and 0.368±0.252, respectively; a quasibinomial distribution was used to account for overdispersion and there was a significant difference between years (t_31_ = −2.087, p = 0.045).

**Figure 5:**
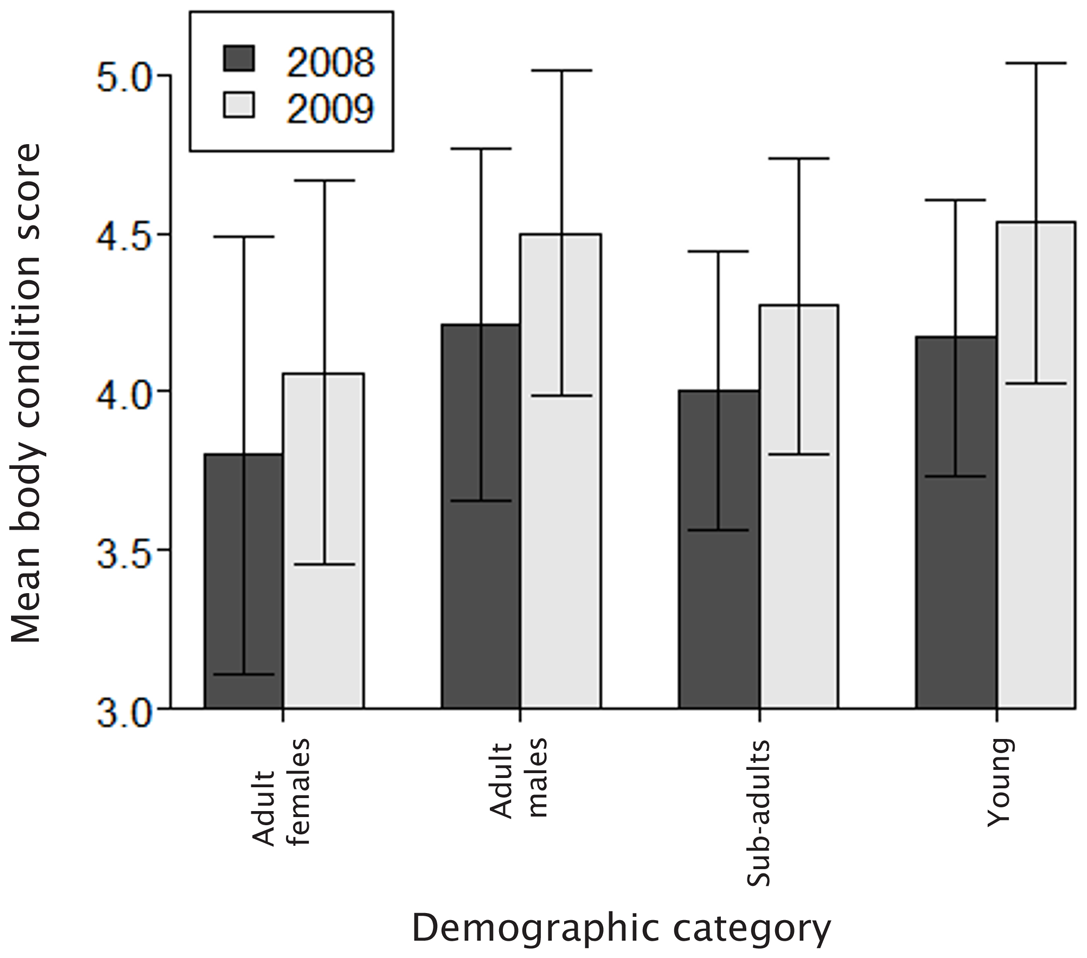
Mean body condition scores of buffalo during the 2008 and 2009 late flood seasons. Error bars represent one standard deviation.

Model simplification showed that BCS was significantly affected by demographic category (LR_3_ = 482.49, p<0.001) and year (LR_1_ = 28.44, p<0.001). Mean BCS in all demographic categories increased in 2009 (Figure 5).

## Discussion

Climatic variability can have a substantial impact on resource availability by altering growth patterns [52], distribution, and relative abundance of plants [53] through species-specific differences in response to changes in water availability and temperature [54]. Both seasonal and annual fluctuations in water levels cause changes in resource availability at the landscape scale. The former are more predictable, and animals adapt to seasonal resource distribution. However, sudden annual changes can disrupt these behaviour patterns by restricting access to critical habitats or by altering productivity [55]. We have shown that stochastic environmental changes in the Okavango Delta cause substantial changes in herbivore behaviour and spatial distribution. The rank order of profitable habitats for buffalo varied temporally through differential responses to seasonal and annual changes in water availability. The proximity of water to particular habitat types was directly related to rainfall and flood levels, which also caused differential vegetation productivity, linked to between-habitat differences in soil type and nutrient content [6]. This combination of water-driven factors resulted in seasonal and annual disparities in habitat selection and associated space use by buffalo. Increased water levels during the 2009 late flood season did not have a significant effect on buffalo behaviour patterns, but reproductive success decreased and body condition increased, highlighting the range of effects caused by stochastic changes in environmental conditions.

### Seasonal Changes in Habitat Selection

Our results supported the hypothesis that buffalo show seasonal differences in habitat selection, with habitats in dry areas selected during the rainy season and those close to permanent water selected during the flood seasons. In the Okavango Delta, buffalo selected habitats with optimal levels of forage biomass and quality in relation to their energetic demands [56], which were higher during the rainy season, when they gave birth and mated, than during the late flood season, when resources were most limiting. Seasonal selection of contrasting habitats enabled buffalo to take advantage of differential profitability [57], while habitats that were avoided benefited from a recovery period due to reduced grazing pressure [58]. The significance of the selection ratios varied seasonally, reflecting changing environmental conditions.

During the rainy season, third order selection was not significant, indicating that buffalo used habitats within their home ranges in proportion to availability [35]. These low selection levels were probably linked to the abundant, high quality forage prevalent across the landscape in the rainy season [2], which reduced the benefit of selective foraging. Second order selection for mopane woodland during the rainy season coincided with increased productivity in that habitat due to the growth of annual grasses [59]. Mopane woodland occurred in dry parts of the buffalos' range, but rainfall in the rainy season created temporary water holes, increasing the accessibility of mopane woodland by removing the spatial constraints imposed by the daily water dependency of buffalo. There were significant differences in selection ratios between the rainy season and both flood seasons, with an emphasis on dry habitats far from permanent water in the former, and habitats close to water channels in the latter.

Overall habitat selection during the early flood season was significant, but mixed acacia woodland, a dry habitat far from permanent water, was the only habitat significantly avoided in second order habitat selection. This was probably because early flood home ranges had to be close to permanent water channels. The three-month delay between the end of the rainy season and vegetative dormancy [60] meant that most forage was still green during the early flood season. Although spatially restricted by water availability, the delayed onset of vegetation senescence during the early flood season meant that buffalo did not need to be as selective in their habitat use as they did during the late flood season, explaining the lack of difference between rainy and early flood season third order selection ratios.

Third order selection was strongest during the late flood season, when vegetation was senescent in most habitats [60], but the receding water caused grasses in both secondary and tertiary floodplains to be at their most productive [61]. The contrast between the profitability of secondary and tertiary floodplains and other habitats resulted in a clumped distribution of favourable resources, and hence a strong selection pressure for those resources [62]. The significant differences between third order selection ratios in the late flood and rainy seasons emphasized the contrast between those two seasons in terms of the rank order of the most favoured habitats. This highlights the strong dependence of buffalo on secondary and tertiary floodplains, which appeared to be acting as resource buffers during the most limiting season [63] by providing access to relatively high quality forage in heterogeneously distributed patches [61].

Seasonal changes in water availability alter the landscape substantially, but animals have adapted to these changes so that they can respond optimally to predictable spatial and temporal fluctuations in resource availability. Such adaptive behaviour has evolved over many generations, and populations may not be able to respond quickly to sudden environmental change [16]. Changes in the timing of seasonal variation in resource availability, a potential result of climate change, could reduce the capacity of animals to identify the most temporally profitable areas and result in e.g. sub-optimal birth periods [64] and altered migration patterns [65]. Access to critical seasonal resources may also be restricted by spatial changes, such as the construction of fences [66] and roads [67], or unusual inundation patterns [17].

### Annual Changes in Habitat Selection

Our results support the hypothesis that changing water levels in the Okavango Delta reduced forage availability in seasonal floodplains, causing buffalo to switch from selecting secondary and tertiary floodplains in 2008 to drier habitats further from permanent water when the floodplains were inundated in 2009. This demonstrated the impact of annual fluctuations in water availability on habitat profitability and selection, which counter-acted to some extent the effects of seasonal water cycles. The time scale of this study was too short for the habitat composition of the range used by the buffalo to change substantially, so the habitat map was valid for both years, but changes in water levels altered the timing and abundance of floodplain forage growth, which was associated with flood waters receding.

The water-dependency of buffalo meant that, in the late flood season, home ranges had to be close to permanent water, which explains the lack of a significant shift in second order selection between the two years. However, the third order selection ratios indicated that buffalo used the habitats within their home ranges differently in 2008 and 2009, spending more time in grassland and riparian woodland than on secondary and tertiary floodplains when water levels were high. The increase in water levels in 2009 was a sudden environmental change, causing large, stochastic variation in resource availability at a landscape scale. Since their productivity was reduced in 2009, the buffering effects of the floodplains were also reduced. To compensate, buffalo had to shift their ranges towards drier habitats, even though these were at their least productive during the late flood season.

### Annual Changes in Buffalo Behaviour, Reproductive Success and Body Condition

Being ruminants, buffalo cannot reduce their resting and ruminating periods below the threshold that allows them to process their forage intake [47], so this restricts their capacity to change their behaviour patterns during periods of low resource availability. While there was some indication that buffalo spent less time resting and more time moving in 2009, when they travelled slightly further on a daily basis, these differences were not significant.

The ratio of young:adult females did not change in 2009, but there was a significant reduction in the proportion of adult females with calves, suggesting a decrease in reproductive success. Calves are the most vulnerable demographic category [68], and would have been the first to suffer mortality in stressful conditions. Buffalo bulls are substantially larger than cows, so have different optimal time budgets, particularly for feeding. Bulls leave breeding herds when their condition falls, forming small temporary bachelor herds in which they forage more intensively [47]. So the lower adult male:adult female ratio in breeding herds in 2009 indicated that environmental conditions were poor. Both these demographic changes confirmed that the reduced abundance of floodplain forage was causing environmental stress for the buffalo, altering herd composition and potentially affecting population dynamics.

However, contrary to our hypothesis, buffalo body condition was significantly higher during the late flood season in 2009 than in 2008; why is unclear. Buffalo utilised ranges further from permanent water during the rest of the year, so the herbaceous layer of areas closer to the channels would have had a lower grazing pressure for most of the year [55], and may still have provided adequate amounts of forage. In addition, there was an unusual rainfall event in 2009, when 60 millimetres of rain fell over June 10–11. The effect on grass growth appears to have been substantial: other herbivores in northern Botswana responded by returning to their rainy season ranges [69], and so this atypical event may have provided more abundant, higher quality forage during the late flood season than was available the previous year.

Periods of low resource availability, such as drought, have a delayed effect on herbivore mortality, only affecting animals in the second year of a prolonged event [15]. While there is little information on the factors influencing fat storage in large mammals [70], herbivores could increase their feeding in the first year of such an event, depleting forage resources but potentially increasing body condition. Delayed mortality would then be a response to prolonged harsh conditions, exacerbated by forage depletion in the previous year. Buffalo may therefore have increased their feeding intake in response to lower forage availability in the floodplains [71], leading to a temporary gain in condition. The large body size of buffalo means that locomotive costs are low, so carrying extra fat would not increase those costs substantially, and any costs would be outweighed by the benefits of having an energetic buffer against future periods of resource deficiency [70]. However, prolonged periods of high flooding in years with typical rainfall patterns would have a detrimental effect on the buffalo, with changes in behaviour, lowered reproductive success, and reduced body condition.

## Conclusions

Rising anthropogenic pressure, both through direct human activities and the effects of climate change, renders environmental conditions less predictable across the globe, and inevitably affects resource availability within protected areas [14], [72]–[74]. Water is one of the most important resources [75] and the future of highly water-dependent riverine and lake ecosystems is uncertain, particularly when several countries lay claim to water extraction rights [23]. The responses of regional ecosystems to environmental change are difficult to predict [76], particularly tropical systems since they are driven by water rather than temperature [1].

Long-term changes in water availability may alter forage availability, thereby affecting the sizes of herbivore home ranges [52] and population dynamics [77], possibly intensifying density-dependent effects [53] and affecting survival and fertility rates [78]. Large-scale climatic fluctuations can also cause significant shifts in habitat selection patterns, ultimately leading to changes in species distributions, potentially causing them to leave protected areas [79], [80]. Substantial resource buffers around seasonally important resources are necessary for herbivores to engage in compensatory behaviours and movements in response to spatial and temporal shifts in resource availability [81]. However, significant changes in environmental conditions may reduce the capacity of animals to exploit these buffers [16]. Since many protected areas are surrounded by human developments [80], the potential for expansion of protected areas is limited [82]. Unusually high water levels can have substantial detrimental impacts [83], and so the management of water resources may be key to the preservation of functioning ecosystems, particularly where animal movements are restricted by barriers. Draining seasonally critical habitats may become necessary to allow herbivores access to productive forage during difficult times of the year and maintain herbivore populations in existing protected areas.
